# Hierarchical functional nanoparticles boost osteoarthritis therapy by utilizing joint-resident mesenchymal stem cells

**DOI:** 10.1186/s12951-022-01297-w

**Published:** 2022-02-19

**Authors:** Yao Lu, Jieli Chen, Lihua Li, Yumei Cao, Yang Zhao, Xiaoyu Nie, Changhai Ding

**Affiliations:** 1 Clinical Research Center, Orthopedic Center, Zhujiang Hospital, Southern Medical University, Guangzhou, Guangdong 510282 China; 2 Guangdong Key Lab of Orthopedic Technology and Implant, General Hospital of Southern Theater Command of PLA, Guangzhou, Guangdong 510010 China; 3 Department of Applied Physics, The Hong Kong Polytechnic University, Hung Hom, Kowloon, Hong Kong, 999077 China; 4 Guangdong Provincial Key Laboratory of Bone and Joint Degeneration Diseases, Academy of Orthopedics, Southern Medical University, Guangzhou, 510630 China; 5 Menzies Institute for Medical Research, University of Tasmania, Hobart, TAS 7000 Australia

**Keywords:** Nanoparticles, Peptide, Osteoarthritis, Joint-resident mesenchymal stem cells, PI3K/AKT/mTOR

## Abstract

Utilization of joint-resident mesenchymal stem cells (MSC) to repair articular cartilage is a promising strategy in osteoarthritis (OA) therapy but remains a considerable research challenge. Here, hierarchical targeting and microenvironment responsive peptide functionalized nanoparticles (NPs) are used to achieve cartilage repair in situ. Ultrasmall copper oxide (CuO) NPs are conjugated with type 2 collagen and MSC dual-targeting peptide (designated WPV) with a matrix metalloproteinase 2 (MMP-2)-sensitive sequence as a spacer to achieve hierarchical targeting. Guided by this peptide, WPV-CuO NPs initially penetrate cartilage and subsequently expose the inner MSC-targeted peptide to attract MSCs through MMP-2 clearance. CuO further promotes chondrogenesis of MSCs. In an anterior cruciate ligament transection rat model, intraarticular injection of WPV-CuO NPs induces significant reduction of cartilage destruction. The therapeutic mechanism involves inhibition of the PI3K/AKT/mTOR pathway, as determined via transcriptome analysis. In conclusion, a novel therapeutic strategy for OA has been successfully developed based on localized MSC recruitment and cartilage repair without transplantation of exogenous cells or growth factors.

**Graphical Abstract:**

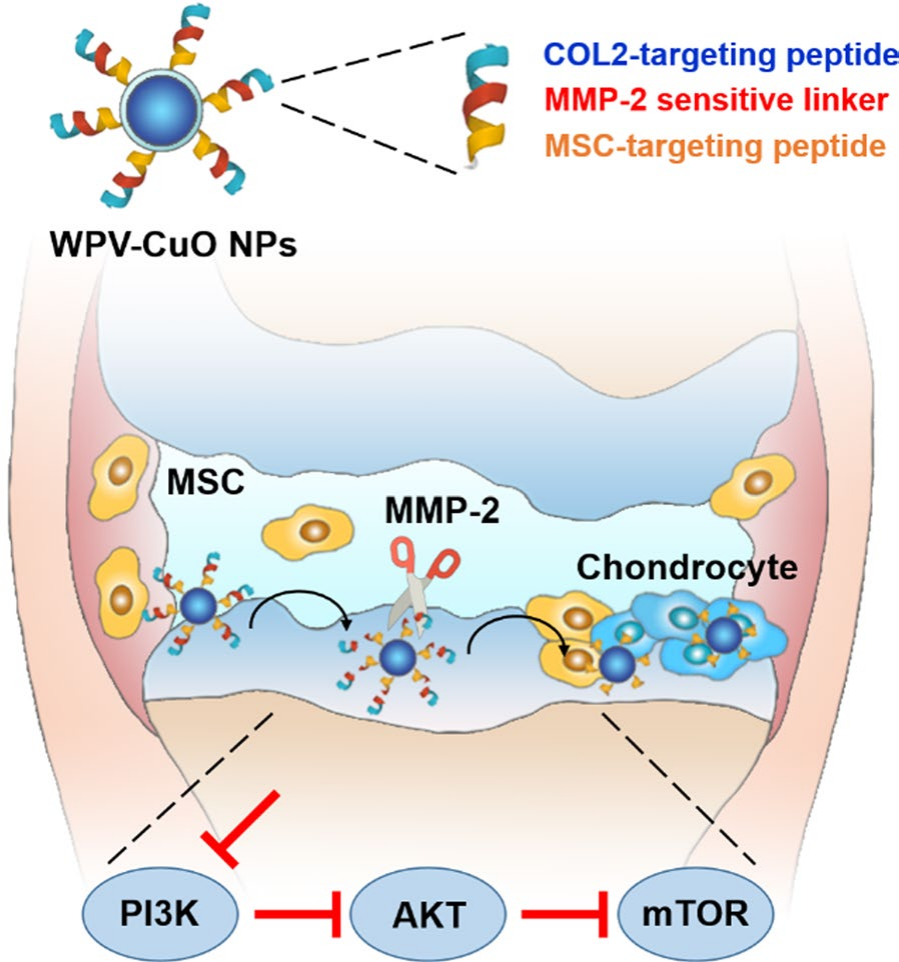

**Supplementary Information:**

The online version contains supplementary material available at 10.1186/s12951-022-01297-w.

## Introduction

Osteoarthritis (OA), the most common degenerative joint disease and the leading cause of adult disability, affects 250 million people worldwide [1]. With the quickening of the aging population process, the rising prevalence of OA will no doubt increase the burden with the disease, becoming a huge global public health challenge for the coming decades [2, 3]. Unfortunately, current pharmacological recommendations for OA therapy are restricted, thus the FDA distinguishes OA as a “serious disease with an unmet medical need” [4].

Analgesic (e.g. paracetamol) and non-steroidal anti-inflammatory drugs (NSAIDs) are commonly used in OA patients for pain reduction and function improvement. However, these drugs provide limited long-term efficacy but raise the risk of gastrointestinal, cardiovascular, liver, and renal adverse events [5–7]. Intraarticular administrations, such as corticosteroids and hyaluronic acid, are also wildly used for the treatment of knee OA to reduce risks of systemic adverse events. However, the effects of intraarticular treatments on symptom relief and structural modification are still uncertain due to the considerable heterogeneity in clinical investigations [8–10]. Cartilage degradation is the most important hallmark of OA, and thus it is a major therapeutic target for the development of disease-modifying OA drugs (DMOADs) [4]. In recent years, sprifermin, a recombinant human fibroblast growth factor 18 (rhFGF18), emerges as a promising DMOAD [11]. Intraarticular injection of sprifermin shows a tendency of positive effects on reduction of cartilage loss, but the clinical significance is still undetermined due to the need of long follow-up periods to detect an effect on joint destruction [12–14]. Other potential DMOADs, such as vitamin D [15], anti-nerve growth factor neutralizing antibodies (e.g. tanezumab) [16], and transforming growth factor-β [17], have been intensively investigated but not used in clinical OA treatment. Overall, current pharmacological interventions mainly alleviate the symptoms temporarily but fail to reverse the basic pathology of OA; namely clinical used drugs are not efficient enough to repair damaged articular cartilage.

Although it is generally believed that damaged cartilage cannot regenerate due to limited endogenous cells [18], mesenchymal stem cells (MSC) are relatively abundant within the joint regions, including synovium, synovial fluid, and adipose tissue [19], that potentially participate in cartilage repair during OA progression [19–21]. In clinic, microfracture surgery is applied to enhancing chondral resurfacing by taking advantage of bone marrow MSCs stimulation [22]. However, bone marrow-resident MSCs only start a chondrogenesis program when bone fracture occurs in adult [23]. Further, microfracture often results in the formation of fibrocartilage with weaker biochemistry and biomechanism rather than natural hyaline articular cartilage [24]. Hence an efficient and facile OA therapy based on joint-resident MSCs is highly desired but remain a challenge. To our knowledge, almost no efforts have been made to utilize joint-resident MSCs for cartilage damage repair in OA therapy to date despite microfracture surgery [25–27]. We aimed to recruit joint-resident MSCs and induce differentiation into chondrocytes to facilitate repair of articular cartilage using nanoparticles (NPs) without the requirement for surgery and exogenous cell transplantation.

**Fig. 1:**
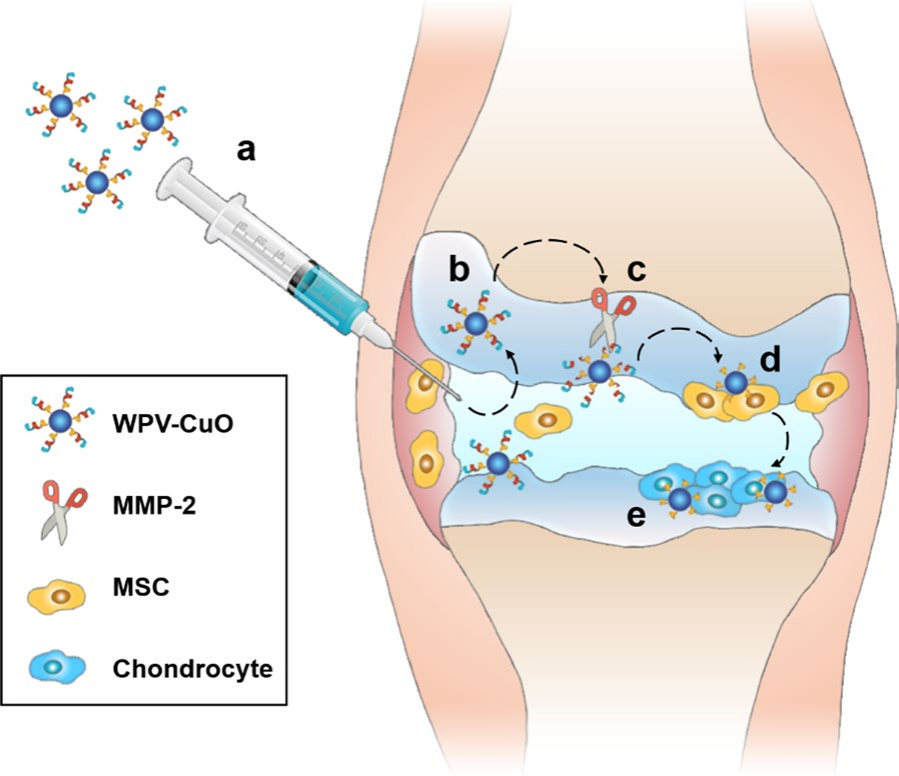
Schematic illustration of application of WPV-CuO NPs in OA therapy. Ultrasmall CuO NPs were modified with dual-targeted (COL2 favoring cartilage penetration and MSC favoring MSC recruitment) peptides, which were spaced with an MMP-2-sensitive sequence. After intraarticular injection into OA joint (**a**), WPV-CuO NPs actively targeted cartilage. **b** Abundant MMP-2 in the OA microenvironment cleared the outer COL2-targeting sequence (**c**) and exposed the inner MSC-targeting sequence to attract joint-resident MSCs (**d**). Directed by CuO NPs, MSCs could differentiate into chondrocytes (**e**), leading to successful repair of cartilage and reduction in joint destruction

Toward this goal, here we prepared ultrasmall copper oxide (CuO) NPs and functionalized them with a hierarchical targeting peptide. We choose Cu-based NPs because earlier studies by our group on Cu-containing materials have demonstrated that Cu promotes chondrogenesis of MSCs and enhances cartilage formation [28, 29]. The functional peptide (designated WPV) was designed to be type 2 collagen (COL2) and MSC dual-targeted (favoring cartilage penetration and MSCs recruitment, respectively), and a matrix metalloproteinase 2 (MMP-2)-sensitive sequence was inserted in the peptide as a spacer to achieve OA-related microenvironment responsive. The resultant WPV-CuO NPs were administered to anterior cruciate ligament transection (ACLT) rat model by intraarticular injection. This peptide-guided NPs could actively target cartilage, followed by its breakdown by abundant MMP-2 in the OA microenvironment, leading to exposure of the inner MSC-targeting peptide for MSC recruitment. Subsequently, the recruited MSCs were chondrogenically induced into chondrocytes by CuO NPs, achieving an efficient OA therapy via cartilage regeneration (Fig. 1).

## Materials and methods

### Preparation of CuO NPs

We synthesized CuO NPs according to a previously reported method with modifications [30]. Briefly, CuCl_2_ solution (10 mM, 50 mL) was heated to 80 °C for 10 min and lascorbic acid aqueous solution (100 mM, 50 mL) added with constant stirring. The pH of the mixture was adjusted to 8.0–9.0 using NaOH solution (1 M) and stirred at 80 °C for 12 h. The resultant solution was centrifuged and dialyzed to obtain CuO NPs.

### Preparation of hierarchical targeting CuO NPs

For carboxyl group modification, 10 mg CuO NPs were reacted with 10 mg polyacrylic acid (PAA) at 60 °C for 2 h under stirring. The resultant PAA-CuO (CuO-COOH) NPs were dialyzed against ddH_2_O to remove excess PAA.

To prepare hierarchical functional NPs, a dual-targeted (COL2 and MSC) and MMP-2-cleavable peptide (WPV, see Additional file 1: Table S1) was designed and conjugated with CuO-COOH NPs at a ratio of 1:10 via *N*’-ethylcarbodiimide hydrochloride/N-hydroxysuccinimide (EDC/NHS) reaction. A control peptide that could not be cleaved by MMP-2 (WGV, see Additional file 1: Table S1) was used to prepare control NPs (termed WGV-CuO) using the same method. Another non-cartilage control peptide (LPV, see Additional file 1: Table S1) was used to prepare LPV-NPs. An NH_2_-rich linker (GGGGKKKK) was added to the C-terminal end of the peptide to facilitate cross-linking with CuO-COOH NPs. The conjugation rate of the peptide was measured by a BCA protein assay kit.

### Characterization of NPs

The shapes and sizes of NPs were examined via high-resolution transmission electron microscopy (HRTEM) and dynamic light scattering (DLS). X-ray diffraction (XRD) was employed to validate the crystalline structure. Changes in zeta potential, Fourier-transform infrared (FTIR) spectra and UV-vis absorption spectra were evaluated. High-performance liquid chromatography (HPLC) was performed to characterize MMP-2 sensitivity.

### Cytotoxicity

Rat synovial MSCs were purchased from Shanghai Zhong Qiao Xin Zhou Biotechnology Co., Ltd. (China) and cultured in MSC medium (Shanghai Zhong Qiao Xin Zhou Biotechnology Co., Ltd.). To examine the cytotoxicity of NPs, MSCs were co-cultured with various concentrations of CuO NPs for 24 h. The cell counting kit-8 (CCK-8) was used for evaluation of cell viability.

### MSC targeting

SMSCs were co-cultured with 100 µg mL^−1^ Cy5.5-labeled WPV-CuO or WGV-CuO NPs and recombinant MMP-2 protein (30 ng mL^−1^; PeproTech, USA). After co-culture for 24 h, MSCs were washed with PBS to remove free CuO NPs and nuclei stained with 4′,6-diamidino-2-phenylindole (DAPI; Sigma, USA). MSCs were examined under a fluorescence microscope (DMI4000, Leica) and analyzed with a flow cytometer (BD FACSCalibur, BD, USA).

### Chondrogenesis

To assay chondrogenic gene expression, MSCs were prepared as above and cultured with chondrogenic medium (Cyagen Biosciences, USA) for 7 days. Relative mRNA expression levels of SOX6, aggrecan (ACAN), and COL2A1 were quantitatively determined via real-time polymerase chain reaction (RT-qPCR). Relative mRNA levels were normalized to that of β-actin using the 2^−△△CT^ method. Primer sequences are described in Additional file 1: Table S2.

MSCs cultures were used for analysis of the chondrogenic inductivity of NPs. Briefly, 4 × 10^5^ MSCs were centrifuged for 5 min at 500 g in a 15 mL tube and cultured for 24 h with chondrogenic medium to obtain a cell pellet, followed by the addition of NPs and MMP-2. After 24 h of culture, the medium was replaced with fresh chondrogenic medium. After 21 days, MSC pellets were fixed in 4% paraformaldehyde and embedded in epoxy resin for histological sectioning. Expression of COL2A1 was detected via immunohistochemistry.

### Cartilage targeting

Male Sprague-Dawley (SD) rats (250–300 g) were purchased from the Medical Experimental Animal Center of Guangdong Province. To establish the cartilage-targeting ability of our newly generated NPs, Cy5.5-labeled WPV-CuO NPs (100 µL, 1 mg mL^−1^) were intraarticularly injected into the knee joint of rats. After 1, 4, and 24 h, rats were euthanized and cartilage specimens from the knee joint were obtained. PBS and Cy5.5-labeled LPV-CuO NPs (100 µL, 1 mg mL^−1^) was administered to the control group. Samples were fixed and decalcified in ethylenediaminetetraacetic acid decalcifying solution (Leagene, China) for 6 weeks. Paraffin-embedded sections were prepared using a routine procedure followed by staining with DAPI. Finally, sections were imaged under a fluorescence microscope (BX51, Olympus, Japan).

### Therapeutic effects of NPs on ACLT rats

An ACLT rat model was established [31] to assess the therapeutic efficacy of NPs against OA in vivo. To establish the abnormal mechanical loading-associated osteoarthritis model, anterior cruciate ligaments of the right knee joint of rats were transected. After 4 weeks, ACLT rats were randomly divided into three treatment groups (n = 6 per group): (1) WPV-NPs, (2) WGV-NPs, and (3) PBS. NPs (100 µL, 1 mg mL^−1^), and PBS (100 µL) were intraarticularly injected into the right knee of ACLT rats following a q7dx4 course (4 times at intervals of 7 days). Sham-operated rats (performed by opening the joint capsule) were used as a control group. Rats were sacrificed at 8 weeks post operation and the right knee joints collected. Samples were fixed, decalcified, and stained with hematoxylin and eosin (H&E) and safranin O-Fast green. MMP-13 and COL2A1 levels were detected via immunofluorescence staining. Additionally, H&E staining of the main organs of rats was performed to evaluate the in vivo biosafety of NPs.

### Biodistribution of WPV-CuO NPs

To investigate the accumulation of WPV-CuO NPs in knee and their metabolism in vivo, Cy5.5-labeled WPV-CuO NPs were intraarticularly injected into the right knees of the OA rats. At day 1, 3, 5 and 10 post-injections, the main organs and knees of the OA rats were collected and imaged using a fluorescence imaging system (Bruker) at Ex: 630 nm, Em: 700 nm.

### Therapeutic mechanisms of action of WPV-CuO NPs against OA

To explore the mechanisms underlying the therapeutic effects of WPV-CuO NPs, transcriptome analysis of ACLT rats was conducted. Established ACLT rats (4 weeks post operation) were randomly divided into two groups (n = 3 per group), specifically, WPV-CuO NPs and PBS, using the same treatment schedules specified above. After a 4-week treatment period, cartilage from the right knee joints was collected and total RNA extracted using TRIzol Reagent. The quality and quantity of RNA samples were determined with Agilent 2100 BioAnalyzer and Qubit RNA assay kits, respectively. mRNA libraries were generated by CapitalBio Technology Co., Ltd (Beijing, China) using Illumina HiSeq X10 (Illumina, USA) according to the manufacturer’s instructions. The raw sequencing quality of each sample was determined with FastQC (v0.11.2) and Fastp (v0.14.0) programs. Tophat (v2.0.13) and Hisat2 (v2.1.0) software was utilized to map the trimmed sequence into the reference genome. Expression levels and differentially expressed genes (DEG) were determined via pairwise comparisons using the R statistical package software limma (v3.32.10). For functional and pathway enrichment analyses, DEGs with fold change ≥ 2, p < 0.05 and false discovery rate (FDR) < 0.05 were selected. KEGG functional enrichment analysis was performed using KOBAS (v3.0) to identify DEGs enriched in the pathway with significant features (p < 0.05).

### Statistical analysis

In vitro data are expressed as mean ± standard deviation (SD) and in vivo data as mean ± standard error of the mean (SEM). The two-sided Student’s t-test was used for analyses.

## Results and discussion

### Design, preparation, and characterization of the NPs

**Fig. 2:**
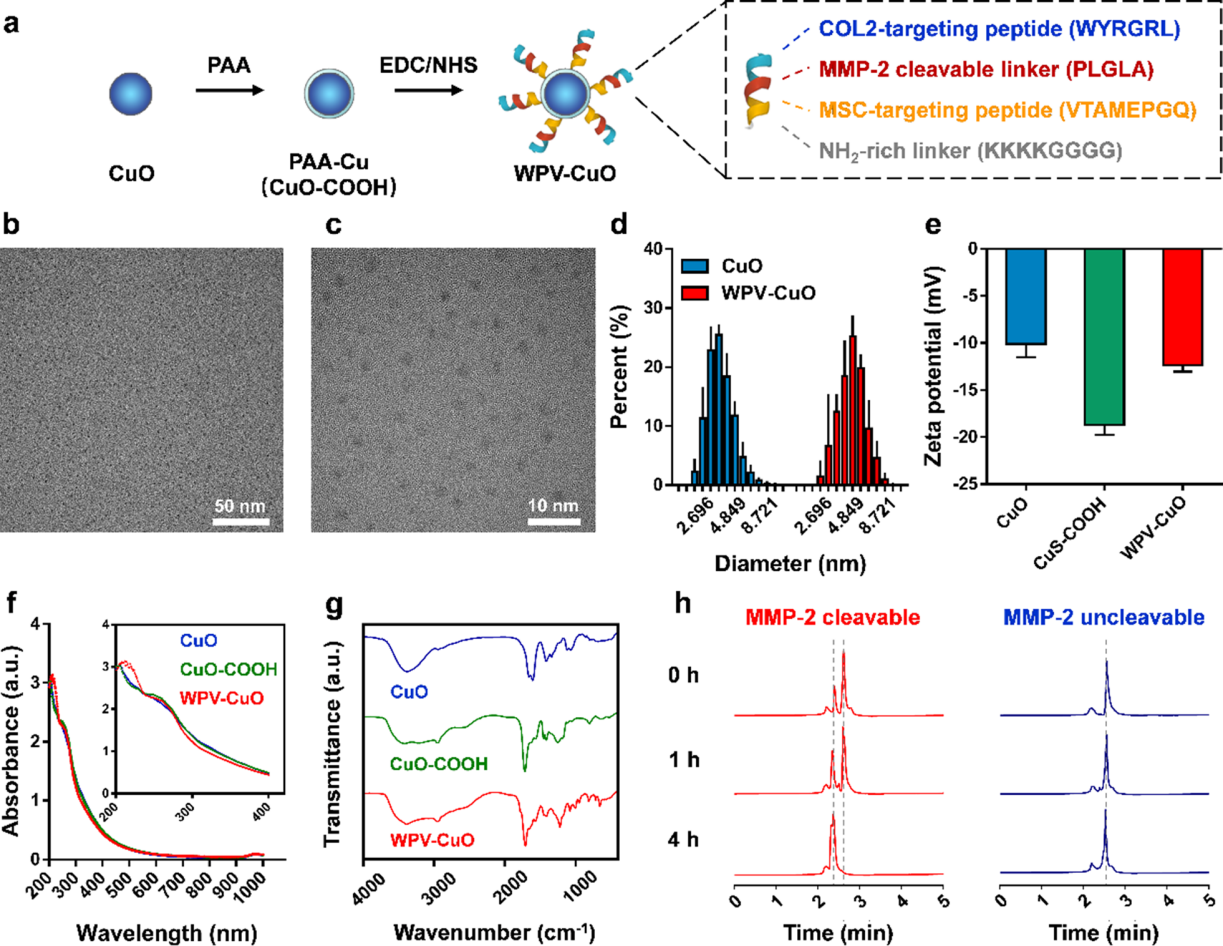
Preparation and characterization of WPV-CuO NPs. **a** Schematic diagram of WPV-CuO generation. Chemically synthesized CuO NPs were coated with PAA to obtain CuO-COOH NPs. A functional peptide (termed WPV) consisting of an outer COL2-targeting sequence (WYRGRL), intermediate MMP-2-sensitive sequence (PLGLA), inner MSC-targeting sequence (VTAMEPGQ), and NH_2_-rich linker was conjugated with CuO-COOH NPs via EDC/NHS reaction to obtain WPV-CuO NPs. **b**, **c** TEM images of CuO NPs. Hydrodynamic diameter distribution (**d**), Zeta potential (**e**), UV–vis spectra and FTIR spectra (**g**) of NPs. Bars represent mean ± SD (*n* = 3). **h** HPLC traces of WPV (MMP-2 cleavable) and WGV (MMP-2 uncleavable control) peptides treated with 30 ng mL^−1^ MMP-2. Peaks of the WPV peptide changed with time while no changes were observed for the WGV peptide, supporting MMP-2 sensitivity of the WPV peptide

In our design, NPs should first penetrate cartilage and subsequently recruit MSCs for repair. Given that NPs larger than 96 nm can barely penetrate the cartilage surface [32], we initially synthesized ultrasmall CuO NPs ~ 3 nm in size (determined via TEM and DLS; Fig. 2b–d). NPs were modified with polyacrylic acid (PAA) to obtain CuO-COOH NPs. To achieve hierarchical functionality, we combined a COL2-targeting peptide (WYRGRL) [32] with a MSC-targeting peptide (VTAMEPGQ) [33] to generate a dual peptide, which was further spaced with the matrix metalloproteinase 2 (MMP-2) clearance sequence PLGLAG [34], designated WPV (Fig. 2a). Another dual-targeting peptide spaced with an adverse version of the MMP-2 sensitive sequence (GALGLP) was generated as an MMP-2 non-sensitive control, termed WGV (Additional file 1: Table S1). An NH_2_-rich linker, GGGGKKKK, was added at the C-terminal end of the peptide to facilitate cross-linking with COOH groups on NPs [35]. Finally, peptides were conjugated with CuO-COOH NPs via an *N*′-ethylcarbodiimide hydrochloride/*N*-hydroxysuccinimide (EDC/NHS) reaction to obtain WPV-CuO and WGV-CuO NPs, respectively.

The size of the NPs slightly increased after peptide modification (Fig. 2d). Zeta potential of the NPs was decreased after COOH groups modification and then it was increased but remained negative after conjugated with the cationic peptides (Fig. 2e). Moreover, WPV-CuO exhibited a new absorption peak at ~ 210 nm compared to CuO and CuO-COOH NPs (Fig. 2f). Fourier transform infrared (FTIR) spectroscopy revealed that a new peak appeared in WPV-CuO NPs at 1568 cm^−1^, which corresponded to amide group in the peptide (Fig. 2g) [35]. These results confirmed successful conjugation of peptides to CuO NPs. The conjugation rate of the peptide on CuO NPs was approximately 94% (determined by a BCA method).

Peptides were further incubated with 30 ng mL^−1^ MMP-2 to stimulate the OA microenvironment and validate MMP-2-mediated breakdown [36]. High-performance liquid chromatography (HPLC) results confirmed cleavage of the MMP-2-sensitive WPV peptide in a time-dependent manner (Fig. 2h). In contrast, the peak intensity of MMP-2-insensitive WGV peptide was not changed in the presence of MMP-2, indicating that the negative control peptide is not cleavable by MMP-2.

### Hierarchical functions of the NPs

**Fig. 3:**
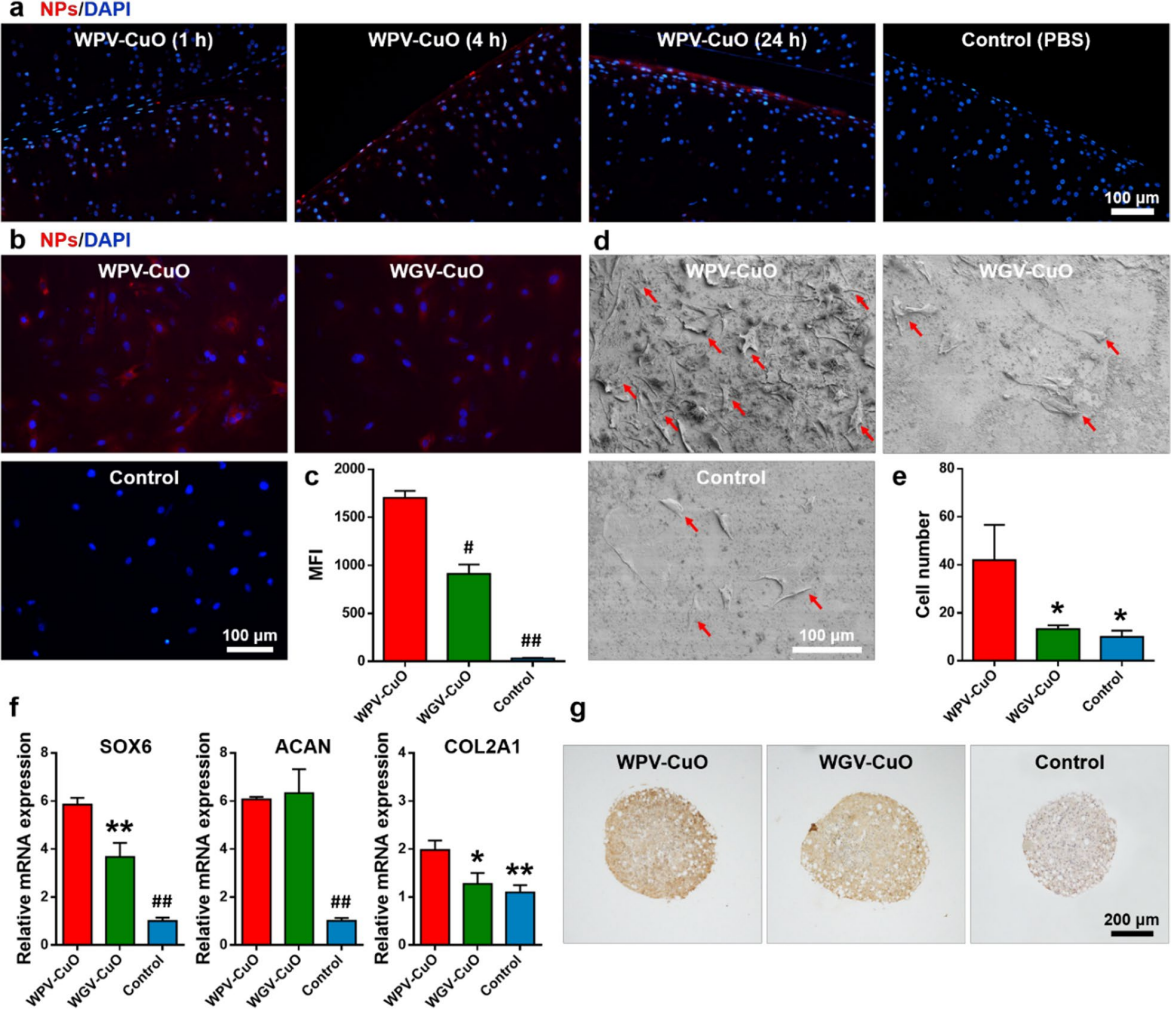
Dual targeting, SMSC recruitment, and chondroinductivity of NPs. **a** Fluorescence images of rat knee joint sections following intraarticular injection of Cy5.5-labled WPV-CuO NPs. WPV-CuO actively penetrated cartilage, even in the presence of MMP-2. Scale bar = 100 μm. **b** Fluorescence images of SMSCs treated with Cy5.5-labeled NPs and MMP-2. Scale bar = 100 μm. **c** Flow cytometry analysis showing highest mean fluorescence intensity (MFI) of SMSCs in the WPV-CuO NP group. Bars represent mean ± SD (*n* = 3). **d** Representative SEM images of SMSC recruitment (highlighted by red arrows) in rat tibial plateau after treatment with NPs and MMP-2. **e** Calculation of the number of SMSCs attached to rat tibial plateau from five random SEM images. The data indicate that WPV-CuO NPs home into cartilage and expose the inner MSC targeting peptide due to MMP-2 sensitivity, leading to significant enhancement of SMSC adhesion to cartilage. Bars represent mean ± SD (*n* = 5). **f** Relative expression levels of chondrogenic genes in SMSCs co-cultured with NPs. Bars represent mean ± SD (*n* = 3). **g** Immunohistological staining of COL2 in SMSC cell pellets co-cultured with NPs. Scale bar = 200 μm. *p < 0.05, **p < 0.01, #p < 0.05, ##p < 0.01

To test the active cartilage-targeting effect of WPV-CuO NPs, Cy5.5-labeled WPV-CuO NPs (100 µg mL^−1^) and MMP-2 were intraarticularly injected into the knees of rats. NPs (red fluorescence) gradually accumulated in cartilage of the knee joints in a time-dependent manner (Fig. 3a). NPs clearly penetrated cartilage tissue after 4 h post-injection and remained distributed within the cartilage matrix after 24 h. In contrast, red fluorescence was negligible in the cartilage after 24 h of intraarticular injection of the non-cartilage-targeting LPV-CuO NPs (Additional file 1: Fig. S1). These results demonstrate that WPV-CuO NPs actively target cartilage before clearance of the COL2-targeting peptide by MMP-2 in vivo.

Next, we examined whether the inner MSC-targeting sequence is effectively exposed after clearance of the MMP-2-sensitive linker. Synovial MSCs (SMSC, the main type of joint-resident MSCs involved in cartilage repair) [19] were co-cultured with Cy5.5-labeled WPV-CuO and WGV-CuO NPs in the presence of MMP-2, respectively. Notably, strong red fluorescence was observed in SMSCs co-cultured with WPV-CuO NPs (Fig. 3b) while red fluorescence was weak in the WGV-CuO NP group. Flow cytometry analysis further confirmed significantly higher fluorescence intensity of WPV-CuO relative to the WGV-CuO and control groups (Fig. 3c; Additional file 1: Fig. S2).

### MSCs recruitment by the NPs

Rat tibial plateau specimens were collected and co-cultured with WPV-CuO or WGV-CuO NPs in the presence of MMP-2. After 24 h, SMSCs were seeded on tibial tissues and co-cultured for an additional 24 h. Large numbers of SMSCs (red arrows) were attached on tibial plateaus treated with WPV-CuO NPs (observed via scanning electron microscopy (SEM); Fig. 3d) while limited SMSCs were detected in WGV-CuO NP and control groups (Fig. 3e). Our findings suggest that WPV-CuO NPs enhance SMSC recruitment through exposure of the inner MSC targeting sequence after breakdown of the MMP-2-sensitive linker. In contrast, WGV-CuO NPs could not attract SMSCs because the inner MSC targeting sequences were not exposed. Thus, WPV-CuO NPs newly developed in this study can successfully promote MSC recruitment via enhancement of MSC adhesion by the inner targeting sequence [37].

### Chondroinductivity of the NPs

Guidance of MSC differentiation into cartilage after recruitment is essential in stem cell-based therapy for OA. Accordingly, the chondroinductive effect of CuO NPs on SMSCs was examined. To this end, SMSCs were co-cultured with WPV-CuO and WGV-CuO NPs at a non-toxic concentration of 100 µg mL^−1^, as determined via the cell counting kit-8 (CCK-8) assay (Additional file 1: Fig. S3). MMP-2 was added and free NPs removed after 24 h. Levels of all chondrogenic genes, including SRY-box transcription factor 6 (SOX6), aggrecan (ACAN), and alpha-1 type II collagen (COL2A1), were significantly higher in NP groups relative to the control group after 7 days of culture (Fig. 3f). Bare CuO NPs also up-regulated chondrogenic gene expression of SMSCs (Additional file 1: Fig. S4). In particular, expressions of the early chondrogenic markers, SOX6, and cartilage matrix marker, COL2A1, were highest in the WPV-CuO groups. In addition, chondrogenic gene expressions were slightly higher in the WPV-CuO than WGV-CuO group, consistent with the finding that WPV-CuO NPs could effectively target SMSCs. COL2 protein expression in SMSC cell pellets was further investigated via immunohistochemistry (Fig. 3g). Cell pellets co-cultured with WPV-CuO NPs presented a deeper brown color compared to WGV-CuO and control groups, supporting chondroinductivity of WPV-CuO NPs. Our results support the functionality of hierarchical WPV-CuO NPs, which can effectively penetrate cartilage, recruit MSCs after clearance of the MMP-2 sensitive linker, and induce differentiation of MSCs into chondrocytes.

### Therapeutic effect of the NPs against OA

**Fig. 4:**
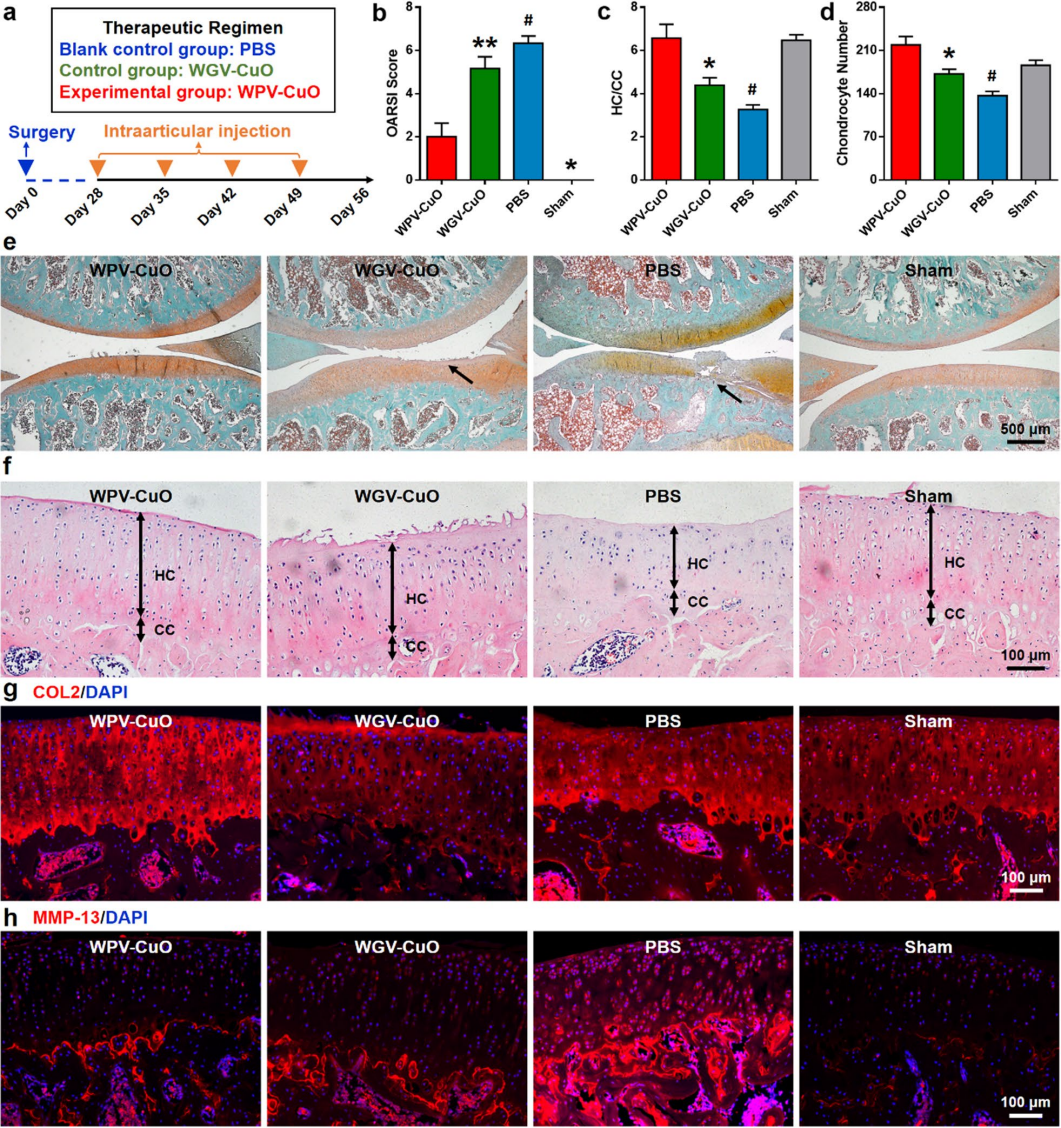
Therapeutic effect of WPV-CuO NPs in an ACLT rat model. **a** Schematic diagram of surgical and different treatments. NPs or PBS were intraarticularly injected into the knee joint 4 weeks after establishment of the ACLT model following a q7dx4 course. A sham treatment group was used as the control. **b** Cartilage erosion evaluation of knee joint sections based on OARSI score. **c** Ratio of hyaline cartilage and calcified cartilage (HC/CC). **d** The number of chondrocytes in the cartilage lesion area. Chondrocytes were calculated from the H&E-stained sections shown in panel **f**. The WPV-CuO NP group presented the lowest OARSI score but highest HC/CC ratio among all treatment groups, indicating that WPV-CuO NPs reduce cartilage damage via generation of hyaline cartilage. Representative safranin O-fast green- (**e**) and H&E-stained (**f**) sections subjected to different treatments. Cartilage erosion is highlighted with black arrows in **d**. Scale bar = 500 μm in d, and = 100 μm in e. Immunofluorescent staining of COL2 (**g**) and MMP-13 (**h**) in cartilage. Bars represent mean ± SEM (*n* = 6). *p < 0.05, **p < 0.01, #p < 0.05, ##p < 0.01

WPV-CuO NPs, WGV-CuO NPs and phosphate buffered solution (PBS) were intraarticularly injected following a q4dx7 course (four times at intervals of 7 days) into knee joints of ACLT rats (Fig. 4a). Notably, WPV-CuO NPs exerted a good therapeutic effect compared to WGV-CuO and PBS groups. Safranin O staining revealed no obvious cartilage damage in WPV-CuO NP-treated ACLT rats (Fig. 4e). In contrast, full-thickness cartilage damage was detected in PBS groups (black arrow). WGV-CuO NPs that lacked the ability to cleave MMP-2 could not expose the MSC targeting sequence but still displayed cartilage homing and chondrogenic properties and also reduced cartilage damage to some extent. Articular cartilage degeneration after different treatments was further graded using the Osteoarthritis Research Society International (OARSI)-modified score (Fig. 4b). The OARSI score of the WPV-CuO group was lowest among all treatment groups and was similar to that of the sham group, indicating that WPV-CuO NPs have the ability to repair cartilage erosion.

Hematoxylin and eosin (H&E) staining disclosed similar thickness of hyaline cartilage (HC) between WPV-CuO NP and sham groups whereas HC was obviously degenerated and thinner in WGV-CuO NP and PBS groups (Fig. 4f). Overall, the hyaline cartilage: calcified cartilage (HC/CC) ratio was significantly decreased in WGV-CuO NP and PBS groups (Fig. 4c). The number of chondrocytes in the cartilage lesion area was significantly decreased in PBS group compared to sham group. Remarkably, the number of chondrocytes increased after WPV-CuO treatment (Fig. 4d). Immunofluorescent staining (Fig. 4g) indicated that COL2 expression was highest in WPV-CuO NP group. Micro-CT scan showed that WPV-CuO NPs slightly inhibited subchondral sclerosis, a typical feature of late-stage OA, compared to PBS group. However, the total bone value and trabecular bone structure had no significant difference between these two groups (Additional file 1: Fig. S5). The results suggest that WPV-CuO NPs exert therapeutic effects against OA and reduce cartilage destruction by promoting generation of hyaline cartilage. MMP13, a key cartilage-degrading enzyme in OA [38], was more highly expressed in WGV-CuO NP and PBS groups compared to WPV-CuO NP and sham groups (Fig. 4h). Data from H&E staining after treatment disclosed no adverse effects of NPs on the main organs of ACLT rats (Additional file 1: Fig. S6).

In addition, we intraarticularly injected Cy5.5-labeled WPV-CuO NPs into OA rats and investigated their metabolism using an in vivo fluorescence imaging system (Additional file 1: Fig. S7). The fluorescence intensity of the right knee gradually decreased over time and WPV-CuO NPs could accumulated in the right knee for approximately 10 days after injection. Moreover, the fluorescence intensity of liver and kidney was stronger than those of the other organs. The results suggest that WPV-CuO NPs are mainly metabolized by the liver and kidney, which is consistent with previous reports that small nanomaterials can be excreted from the body by hepatic metabolism and renal clearance [39, 40].

### Therapeutic mechanisms of the NPs against OA

**Fig. 5:**
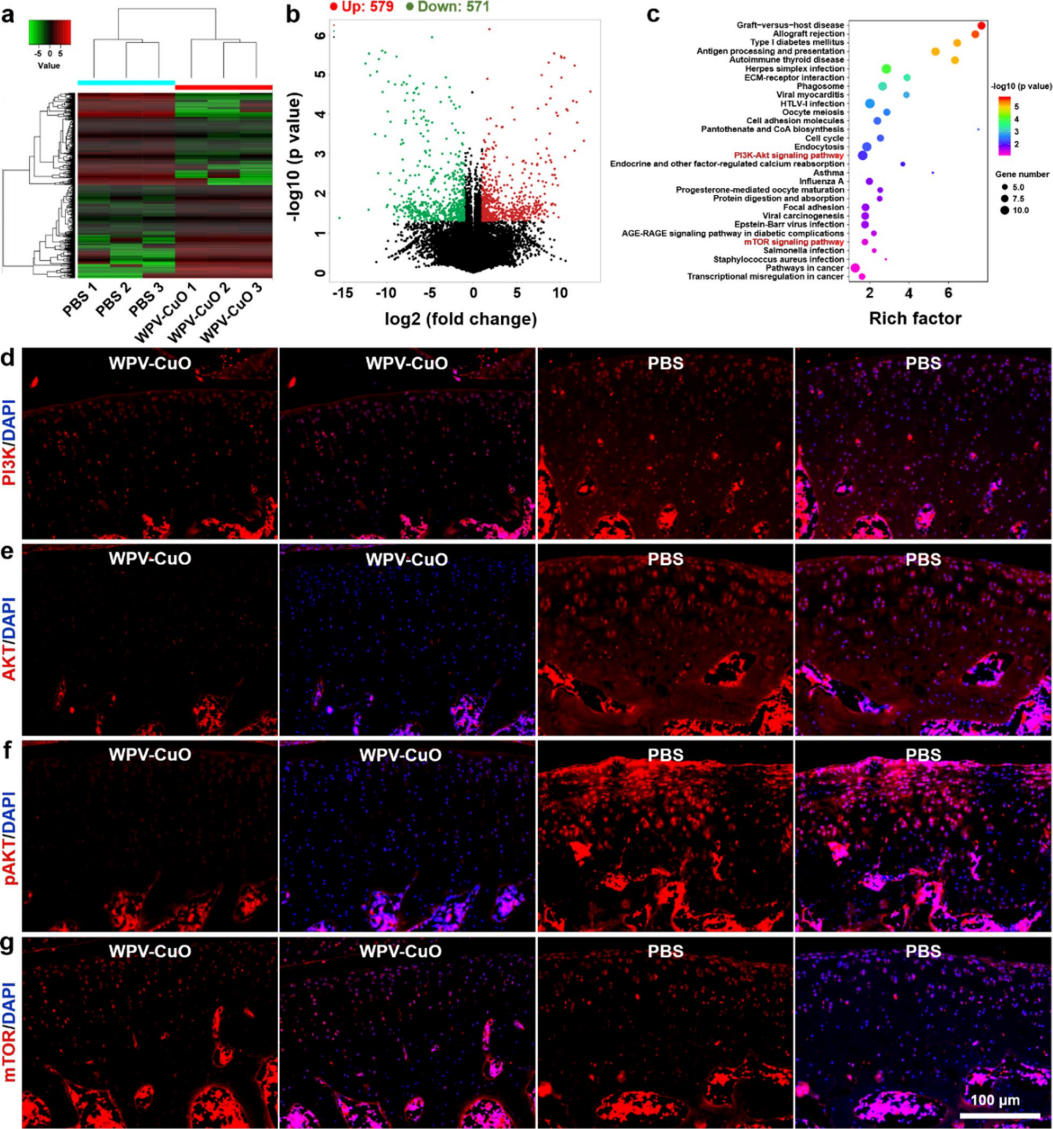
Therapeutic mechanisms underlying activity of WPV-CuO NPs in OA. **a** Heatmap of significantly upregulated and downregulated genes between ACLT rats treated by PBS or WPV-CuO NPs. **b** Volcano plots showing significantly upregulated and downregulated genes following WPV-CuO treatment. Overall, 579 genes were up-regulated and 571 genes were down-regulated. **c** KEGG pathway enrichment analysis of the 30 most significantly enriched pathways. Immunofluorescent staining of PI3K (**d**), AKT (**e**), pAKT (**f**), and mTOR (**g**) expressed in cartilage after treatments. These data indicated that WPV-CuO NPs inhibited PI3K/AKT/mTOR pathway in OA model. Scale bar = 100 μm

To further clarify the mechanisms underlying activity of WPV-CuO NPs against OA, transcriptome analysis of collected cartilage from knee joints of ACLT rats after treatment was performed. Heatmap revealed the significant differentially expressed genes (DEG) between WPV-CuO NP- and PBS-treated ACLT rats (Fig. 5a). Overall, 579 genes were upregulated and 571 genes were downregulated in the WPV-CuO NP group relative to the PBS group (Volcano plots, Fig. 4b).

Kyoto Encyclopedia of Genes and Genomes (KEGG) pathway enrichment analysis suggested that phosphoinositide 3-kinase/protein kinase B (PI3K/AKT) and mammalian target of rapamycin (mTOR) signaling pathways are highly related to the therapeutic mechanisms (Fig. 5c). Previous studies have demonstrated that activation of PI3K/AKT/mTOR enhances MMP production but reduces autophagy of chondrocytes, thereby accelerating cartilage destruction in OA [41–43]. Notably, PI3K and AKT as well as phosphorylated AKT (p-AKT) were significantly decreased in the WPV-CuO NP group, indicating suppression of the PI3K/AKT pathway (Fig. 5d–f). Moreover, downstream mTOR expression was significantly inhibited in the WPV-CuO treatment group (Fig. 5g). Our results are consistent with recent literature reports that MSC transplantation suppresses PI3K/AKT/mTOR to ameliorate tissue injury [44–46]. Therefore, the potential therapeutic mechanism underlying the activity of WPV-CuO NP against OA may be summarized as follows: WPV-CuO NPs recruit joint-resident MSCs and induce differentiation into chondrocytes, promoting repair of damaged cartilage and reduction of cartilage destruction through inhibition of the PI3K/AKT/mTOR pathway.

## Conclusions

In summary, we have developed a facile peptide-modified NP strategy that takes advantage of joint-resident MSCs to achieve efficient OA therapy. The hierarchical functional peptide WPV facilitates active homing to cartilage by CuO NPs, subsequent recruitment of MSCs and chondrogenic induction to repair cartilage in an OA-related MMP-2 abundant microenvironment. WPV-CuO NP-mediated MSC therapy not only reduced joint destruction by generating hyaline cartilage but also inhibited the PI3K/AKT/mTOR pathway, thereby enhancing the therapeutic effect of OA in vivo. Our newly developed NPs provide a general and useful tool for treating many other types of arthritis, since cartilage erosion and presence of MMP-2 are common features of degenerative and inflammatory joint diseases, such as rheumatoid arthritis [47], psoriatic arthritis [48], gouty arthritis [49], and systemic lupus erythematosus [50].

## Supplementary Information

**Additional file 1: Fig. S1. **Cartilage-targeting effect of the control LPV-CuO NPs (red fluorescence). Scale bar = 100 μm. **Fig. S2. **Flow cytometry analysis of SMSCs co-cultured with WPV-CuO, WGV-CuO, and PBS in the presence of MMP-2. **Fig. S3.** Cell viability of SMSCs co-cultured with CuO NPs after 24 h. **Fig. S4**. Chondrogenic inductivity of bare CuO NPs (n=3). **Fig. S5**. Micro-CT scan of the joints of OA rats treated with WPV-CuO or PBS (n=6). **Fig. S6**. H&E staining of the main organs of ACLT rats after different treatments.Scale bar = 500 μm. **Fig. S7**. Biodistribution of the Cy5.5-labeled WPV-CuO NPs after intraarticular injection. **Table S1. **Peptide properties. **Table S2. **Primers sequence used for RT-qPCR.

## Data Availability

All data generated or analyzed during this study are included in this published article and the Supporting Information.
