# Supplementary material for: Hierarchical functional nanoparticles boost osteoarthritis therapy by utilizing joint-resident mesenchymal stem cells
Source: J Nanobiotechnology. 2022 Feb 19;20:89. doi: 10.1186/s12951-022-01297-w (PMC8858465; doi:10.1186/s12951-022-01297-w)
Supplement: Supplementary file 1 — Additional file 1: Fig. S1. Cartilage-targeting effect of the control LPV-CuO NPs (red fluorescence). Scale bar = 100 μm. Fig. S2. Flow cytometry analysis of SMSCs co-cultured with WPV-CuO, WGV-CuO, and PBS in the presence of MMP-2. Fig. S3. Cell viability of SMSCs co-cultured with CuO NPs after 24 h. Fig. S4. Chondrogenic inductivity of bare CuO NPs (n=3). Fig. S5. Micro-CT scan of the joints of OA rats treated with WPV-CuO or PBS (n=6). Fig. S6. H&E staining of the main organs of ACLT rats after different treatments.Scale bar = 500 μm. Fig. S7. Biodistribution of the Cy5.5-labeled WPV-CuO NPs after intraarticular injection. Table S1. Peptide properties. Table S2. Primers sequence used for RT-qPCR. [file 12951_2022_1297_MOESM1_ESM.docx]

Supporting Information

Hierarchical functional nanoparticles boost osteoarthritis therapy by utilizing joint-resident mesenchymal stem cells

Yao Lu^a,b,1,^*, Jieli Chen^a,1^, Lihua Li^c^, Yumei Cao^a^, Yang Zhao^a^, Xiaoyu Nie^a^, Changhai Ding^a,d,e,^*

^a^ Clinical Research Center, Department of Joint and Orthopedics, Orthopedic Center, Zhujiang Hospital, Southern Medical University, Guangzhou, Guangdong 510282, China

^b^ Guangdong Key Lab of Orthopedic Technology and Implant, General Hospital of Southern Theater Command of PLA, Guangzhou, Guangdong 510010, China

^c^ Department of Applied Physics, The Hong Kong Polytechnic University, Hung Hom, Kowloon, Hong Kong 999077, China.

^d^ Guangdong Provincial Key Laboratory of Bone and Joint Degeneration Diseases, Academy of Orthopedics, Southern Medical University, Guangzhou, 510630, China.

^e^ Menzies Institute for Medical Research, University of Tasmania, Hobart, Tasmania 7000, Australia

^1^ These authors contributed equally to this work.

* Corresponding authors: Email: oayul@smu.edu.cn (Y. Lu.), changhai.ding@utas.edu.au (C. Ding.).


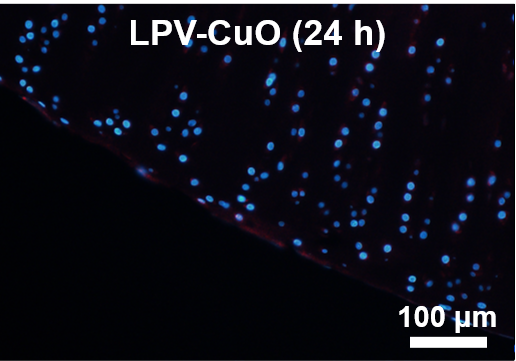


**Figure S1.** Cartilage-targeting effect of the control LPV-CuO NPs (red fluorescence). Scale bar = 100 μm.


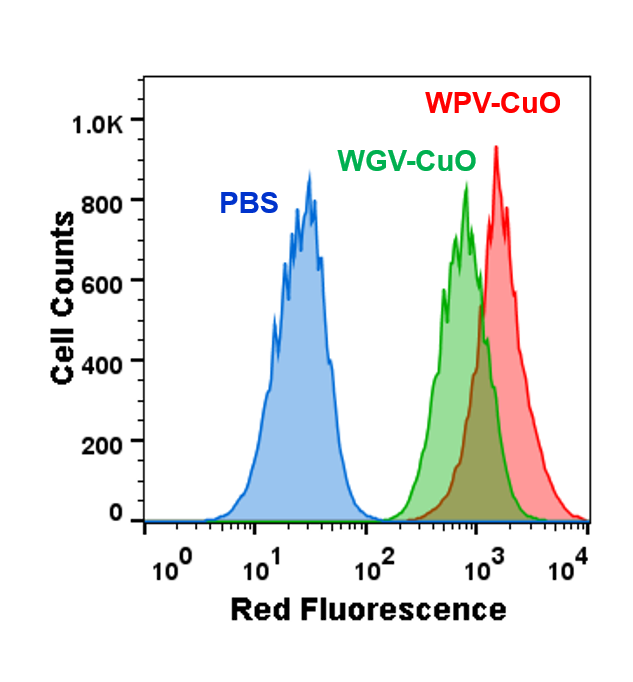


**Figure S2.** Flow cytometry analysis of SMSCs co-cultured with WPV-CuO, WGV-CuO, and PBS in the presence of MMP-2.


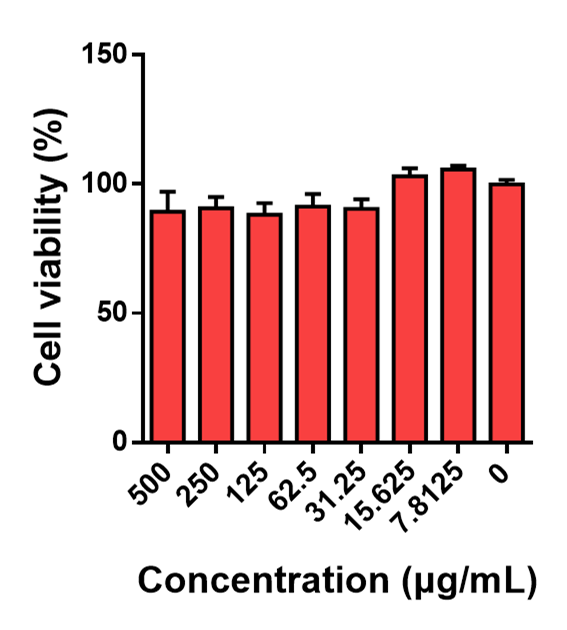


**Figure S3.** Cell viability of SMSCs co-cultured with CuO NPs after 24 h.


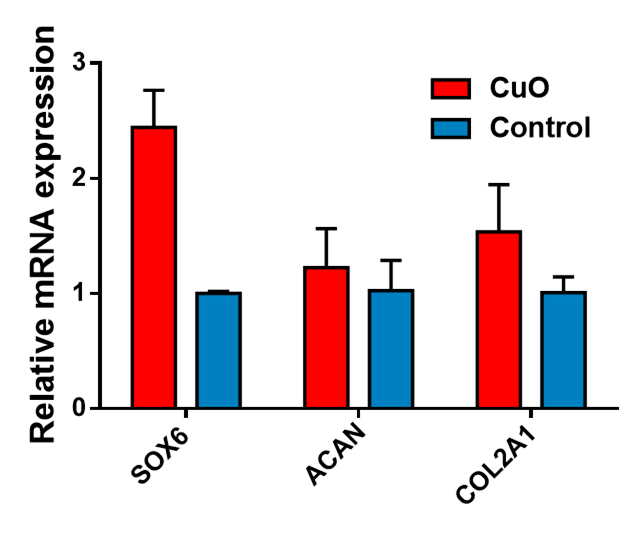


**Figure S4**. Chondrogenic inductivity of bare CuO NPs (n=3).


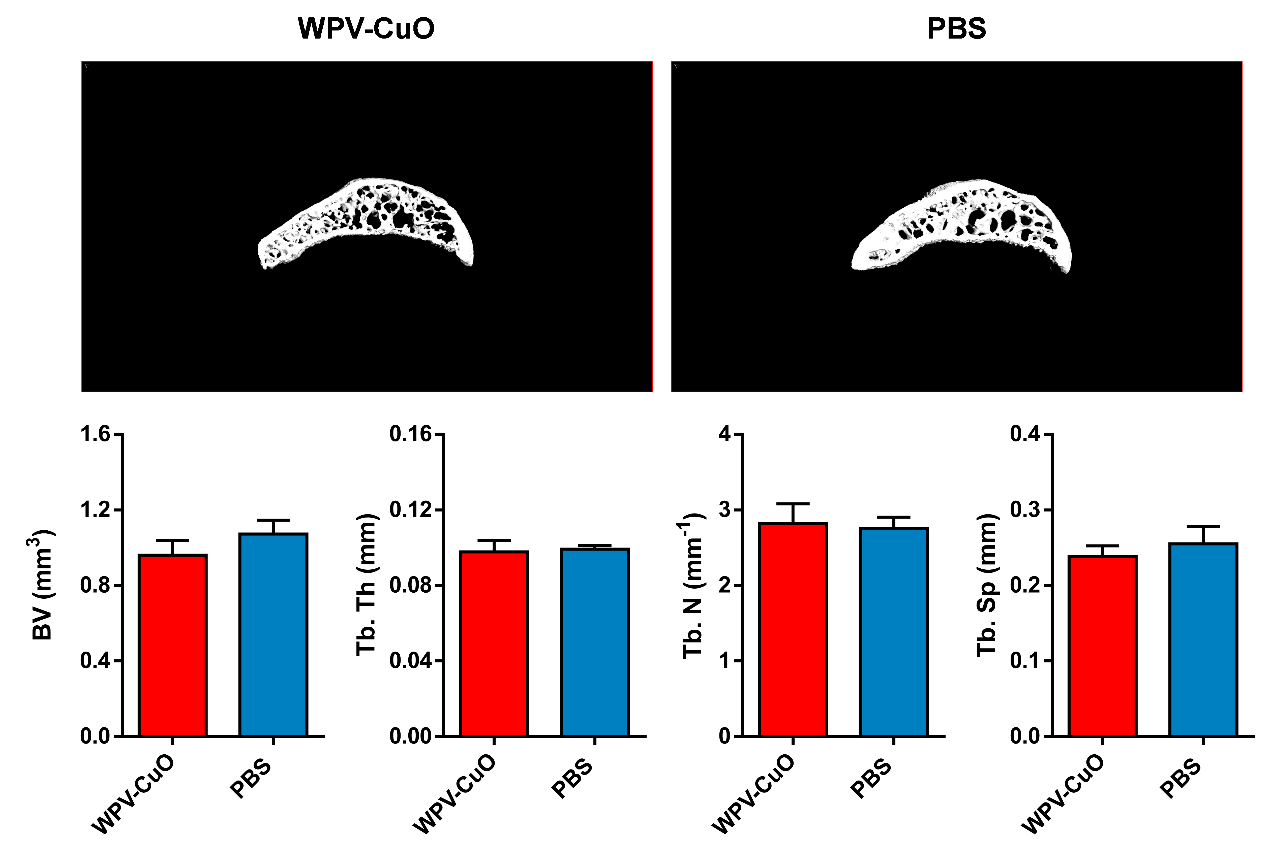


**Figure S5**. Micro-CT scan of the joints of OA rats treated with WPV-CuO or PBS (n=6).


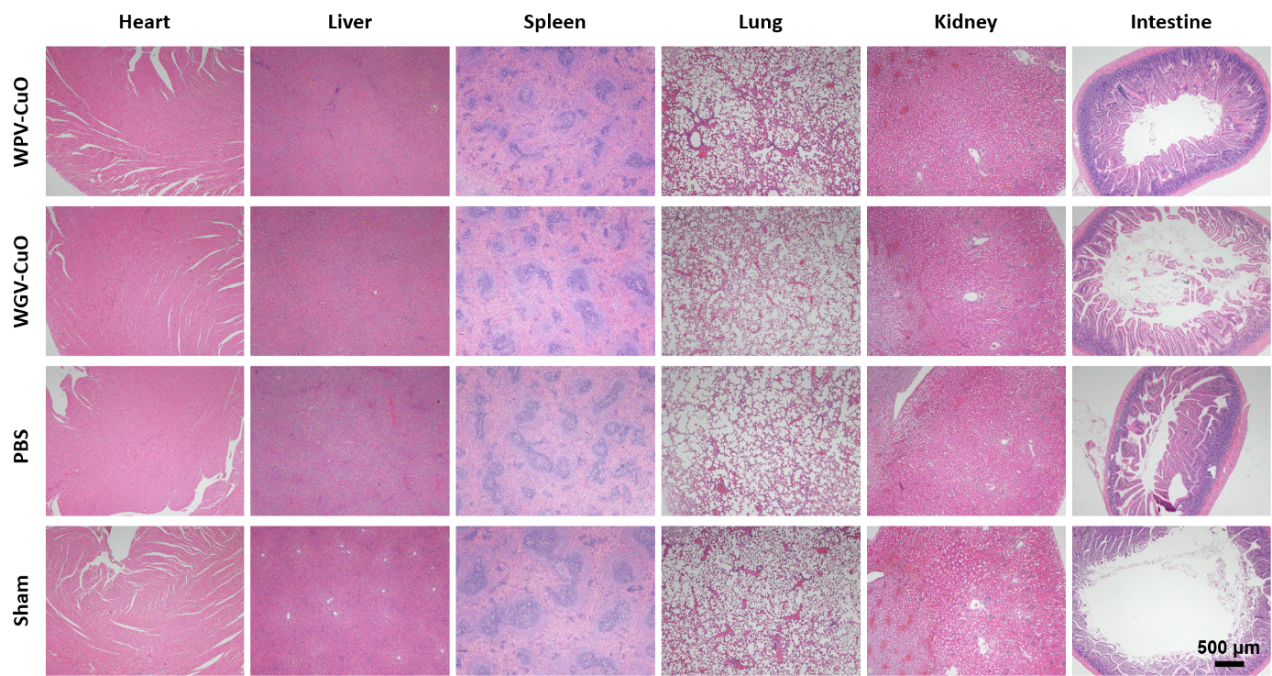


**Figure S6**. H&E staining of the main organs of ACLT rats after different treatments. Scale bar = 500 μm.


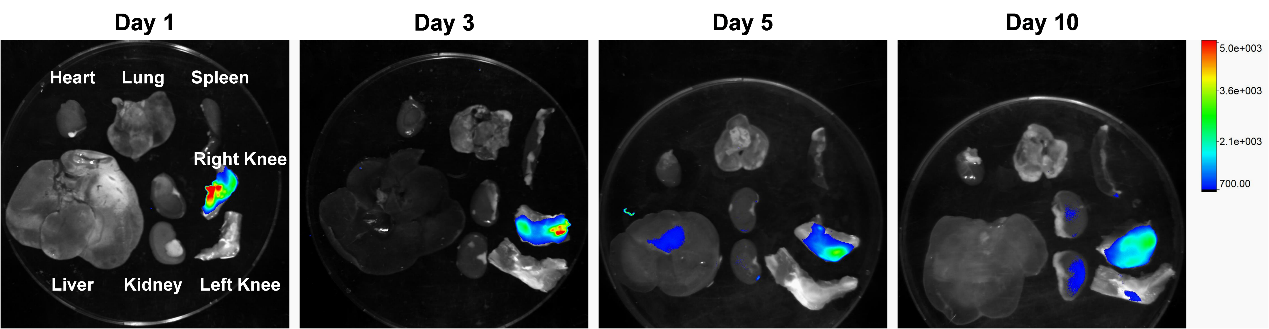


**Figure S7**. Biodistribution of the Cy5.5-labeled WPV-CuO NPs after intraarticular injection.

**Table S1.** Peptide properties.

| **Peptide** | **Sequence** | **Description** |
| --- | --- | --- |
| **WPV** | WYRGRLGGGSPLGLAGGGGSVTAMEPGQGGGGKKKK | COL2- and MSC-targeting,  MMP-2 cleavable |
| **WGV** | WYRGRLGGGSGALGLPGGGSVTAMEPGQGGGGKKKK | COL2- and MSC-targeting,  MMP-2 uncleavable |
| **LPV** | LRGRYWGGGSGALGLPGGGSVTAMEPGQGGGGKKKK | Non-COL2-targeting, MSC-targeting,  MMP-2 uncleavable |

**Table S2.** Primers sequence used for RT-qPCR.

| **Gene** | **Primer sequence** | |
| --- | --- | --- |
|  | **Forward** | **Reverse** |
| **β-actin** | 5′-TATAAAACCCGGCGGCGCA-3′ | 5′-TCATCCATGGCGAACTGGTG-3′ |
| **SOX6** | 5′-CCCTCTGTCACTGTCACGTT-3′ | 5′-ACAGGCAAATGGAGAGGTGG-3′ |
| **ACN** | 5′-TCGAATCCCCAAATCCCTCAT-3′ | 5′-ACATTGCTCCTGGTCTGCAA-3′ |
| **COL2A1** | 5′-GCCAGGATGCCCGAAAA-3′ | 5′-TTGTCACCACGATCACCTCTG-3′ |
